# Constitutive TL1A (TNFSF15) Expression on Lymphoid or Myeloid Cells Leads to Mild Intestinal Inflammation and Fibrosis

**DOI:** 10.1371/journal.pone.0016090

**Published:** 2011-01-11

**Authors:** David Q. Shih, Robert Barrett, Xiaolan Zhang, Nicole Yeager, Hon Wai Koon, Piangwarin Phaosawasdi, Yahui Song, Brian Ko, Michelle H. Wong, Kathrin S. Michelsen, Gislaine Martins, Charalabos Pothoulakis, Stephan R. Targan

**Affiliations:** 1 Inflammatory Bowel and Immunobiology Research Institute, Cedars-Sinai Medical Center, Los Angeles, California, United States of America; 2 Department of Gastroenterology, The Second Hospital of Hebei Medical University, Shijianzuang, China; 3 Division of Digestive Diseases, Inflammatory Bowel Disease Center, David Geffen School of Medicine, University of California Los Angeles, Los Angeles, California, United States of America; Charité, Campus Benjamin Franklin, Germany

## Abstract

TL1A is a member of the TNF superfamily and its expression is increased in the mucosa of inflammatory bowel disease patients. Moreover, a subset of Crohn's disease (CD) patients with the risk *TL1A* haplotype is associated with elevated TL1A expression and a more severe disease course. To investigate the *in vivo* role of elevated TL1A expression, we generated two transgenic (*Tg*) murine models with constitutive Tl1a expression in either lymphoid or myeloid cells. Compared to wildtype (WT) mice, constitutive expression of Tl1a in either lymphoid or myeloid cells showed mild patchy inflammation in the small intestine, which was more prominent in the ileum. In addition, mice with constitutive Tl1a expression exhibited enhanced intestinal and colonic fibrosis compared to WT littermates. The percentage of T cells expressing the gut homing chemokine receptors CCR9 and CCR10 was higher in the *Tl1a Tg* mice compared to WT littermates. Sustained expression of Tl1A in T cells also lead to increased Foxp3+ Treg cells. T cells or antigen presenting cells (APC) with constitutive expression of Tl1a were found to have a more activated phenotype and mucosal mononuclear cells exhibit enhanced Th1 cytokine activity. These results indicated an important role of TL1A in mucosal T cells and APC function and showed that up-regulation of TL1A expression can promote mucosal inflammation and gut fibrosis.

## Introduction

Inflammatory bowel disease (IBD), encompassing CD and ulcerative colitis (UC), is a chronic inflammatory disorder caused by dysregulated immune responses in a genetically predisposed individual (reviewed in [1]). CD is a chronic inflammatory condition that predominately affects the gut with distinctive pathological features such as patchy transmural intestinal inflammation, relative sparing of inflammation in the rectum, intestinal fibrostenosis and a dysregulated T helper (Th) 1 and Th17 immune response [1], [2]. Accumulating data, including genome-wide association studies (GWAS), demonstrate that more than 80 distinct genetic loci confer CD susceptibility, and are being used to define critical molecules and pathways that converge in physiologic processes that lead to mucosal inflammation [2], [3]. Among the several recently discovered IBD associated gene variants, only those in the tumor necrosis factor superfamily member 15 (*TNFSF15*) have been shown to be associated with CD in all ethnic and age groups [1].

TL1A plays an important role in modulating the adaptive immune response. In the Th1 effector immune response, binding of TL1A to its receptor (death domain receptor 3, DR3, TNFRSF25) enhances IFN-γ production from peripheral and mucosal T cells [4], [5]. Studies have shown that TL1A can be induced in APC by FcγR signaling [6], [7] and microbial antigen/organisms [8] suggesting that augmentation of the Th1 immune response by TL1A may occur through APC-T cell interactions. In murine models of chronic mucosal inflammation, TL1A enhanced Th1 and Th17 effector function by up-regulating IFN-γ and IL17 production, respectively, in gut-associated lymphoid tissue (GALT) CD4^+^ cells under Th1/Th17 polarizing conditions, indicating that TL1A is an important modulator in the development of gut mucosal inflammation [9]. In addition to mediating Th1 responses, TL1A also promotes Th2 and Th17 effector cell function [10], [11], [12].

Several studies implicate the TL1A/DR3 signaling pathway in mucosal inflammation. The expression of TL1A and DR3 is up-regulated in the inflamed gut mucosa of two distinct murine models of ileal inflammation [13]. Neutralizing anti-mouse TL1A Ab attenuated inflammation in both the dextran sulfate sodium induced chronic colitis and a G protein αi2-/- (*Gαi2^−/−^*) T cell transfer colitis model [9]. In humans, TL1A can enhance IFN-γ production in CD4^+^ T cells expressing the gut-homing receptor CCR9 [14]. Increased expression of both TL1A and DR3 was found in gut mucosal biopsies and lamina propria (LP) T cells of both UC and CD patients [4], [15]. Furthermore, TL1A and DR3 expression are correlated with severity of gut mucosal inflammation as their transcripts were several times more abundant in RNA from mucosal biopsies taken from inflamed CD lesions than in those taken from uninvolved areas [4], [15].

To determine the *in vivo* consequence of increased TL1A expression, we generated two *Tg* murine models that constitutively express Tl1A in either lymphoid or myeloid cells. We found that constitutive expression of Tl1A in either lymphoid or myeloid cells induced mild spontaneous patchy intestinal inflammation by 10 months of age. We showed that a higher percentage of T cells and APC from the mesenteric lymph nodes (MLN) have an activated phenotype and express the gut homing chemokine receptors CCR9 and CCR10, associated with increased production of IFN-γ. Consistent with previous reports [16], [17], we observed goblet cell hyperplasia and an increased number of Paneth cells in the ileum of *Tl1a* transgenic (*Tg*) mice. We also showed enhanced intestinal and colonic fibrosis in mice that constitutively express Tl1a. These 2 novel murine *Tl1A Tg* models have patterns of site directed mucosal inflammation and fibrosis seen in human CD and may be useful models to study the pathogenesis of IBD.

## Results

### Generation of *in vivo* constitutive Tl1a expression in the myeloid and T cell lineage

**Figure 1:**
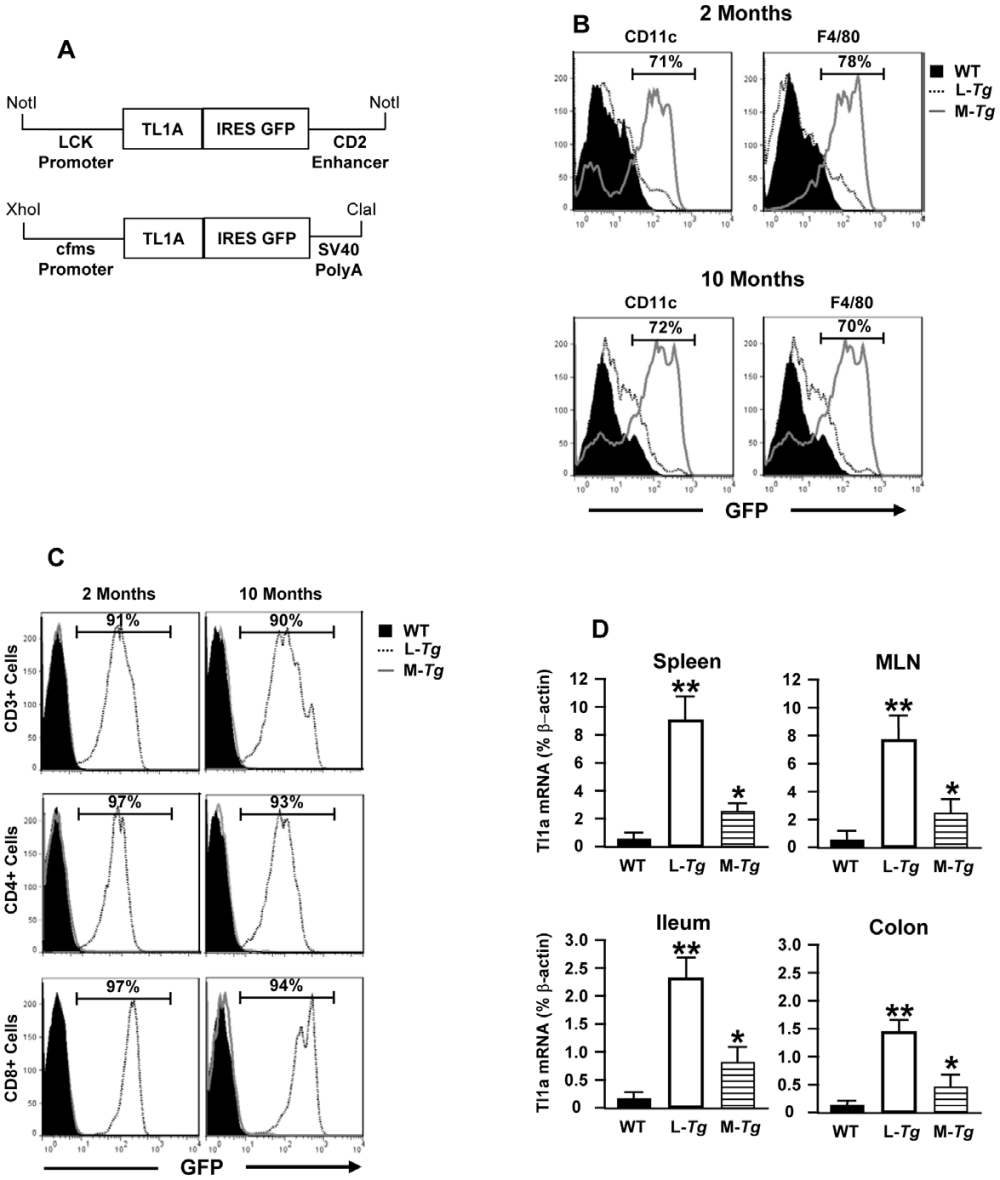
Generation of constitutive *in vivo* expression of Tl1a in T- and antigen presenting cells. (A) Schematic of *LCK-CD2-Tl1a* and *FMS-Tl1a* transgenic construct. An internal ribosomal entry site (IRES) element is used for both transgenic constructs so that a bi-cistronic message can be made from the transgene and the Tl1a expressing cells are tagged by GFP. (B) Flow cytometric analysis of the transgene marker GFP on either CD11c or F4/80 gated splenocytes from WT (black filled), *LCK-CD2-Tl1a Tg* mice (L-*Tg*, dotted line) or *FMS-Tl1a Tg* mice (M-*Tg*, solid grey line). Representative histograms are shown. (C) Representative analysis of the transgene marker GFP on either CD3, CD4 or CD8 gated splenocytes from WT (black filled), *LCK-CD2-Tl1a Tg* mice (L-*Tg*, dotted line) or *FMS-Tl1a Tg* mice (solid grey line). (D) Tl1a mRNA expression was determined in the spleen, MLN, ileum or colon by real-time polymerase chain reaction. Data are expressed as mean percent of β-actin ± standard deviation (SD). *P<0.05, **P<0.01. n = 6 independent littermate mice per group were used for B-D.

To investigate the contribution of sustained APC or T cell Tl1a expression on gut mucosal homeostasis and inflammation, we generated *Tg* mice that constitutively express Tl1a in either T cells or myeloid cells. We used the proximal *lck* promoter and *CD2* enhancer to drive T cell lineage-specific expression and the *c-fms* promoter to mediate myeloid specific expression in APC such as macrophages and DC [18], [19]. We also cloned an *IRES-GFP* element downstream of the murine *Tl1a* so that *Tg* Tl1a expressing cells could be identified by GFP. The cloning strategies and schematic of the *Tg* construct are described in Materials and Methods section and figure 1A, respectively. The *Tg* constructs were injected into C57BL/6 pronuclei to ensure genetic homogeneity except for the *Tl1a* transgene.

Expression of the transgene was determined in myeloid *Tg* mice (named *FMS-Tl1a-GFP Tg*) and T-cell lineage specific expression of Tl1a (named *LCK-CD2-Tl1a-GFP Tg*) by the expression of GFP. We found that in the *FMS-Tl1a-GFP Tg* mice, GFP was present in over 70% of CD11c and F4/80 positive cells, and the expression of the Tl1a transgene persisted as the mice aged (Fig. 1B). The specificity of the *c-fms* promoter was illustrated by the fact that we did not detect GFP expression in CD3, CD4 or CD8 positive T cells (Fig. 1C). In the *LCK-CD2-Tl1A-GFP Tg* mice, GFP was present in over 90% of CD3, CD4 and CD8 positive cells (Fig. 1C). Similar to the *FMS-Tl1a-GFP Tg*, the expression of the transgene in the lymphoid *Tl1a Tg* mice also persisted as the mice got older (Fig. 1C). Less than 10% GFP expression was detected in F4/80 or CD11c positive cells (Fig. 1B), indicating that the *Lck* promoter and *Cd2* enhancer element drives T cell lineage-specific expression. We directly showed that Tl1a mRNA is higher in the spleen, mesenteric lymph nodes (MLN), colon and ileum of both *Tg* mice compared to WT mice (Fig. 1D). Together, these data demonstrate that we generated tissue specific constitutive *in vivo* expression of Tl1a in APC and T cells.

### Mice with constitutive Tl1a expression do not develop gross tissue inflammation

**Figure 2:**
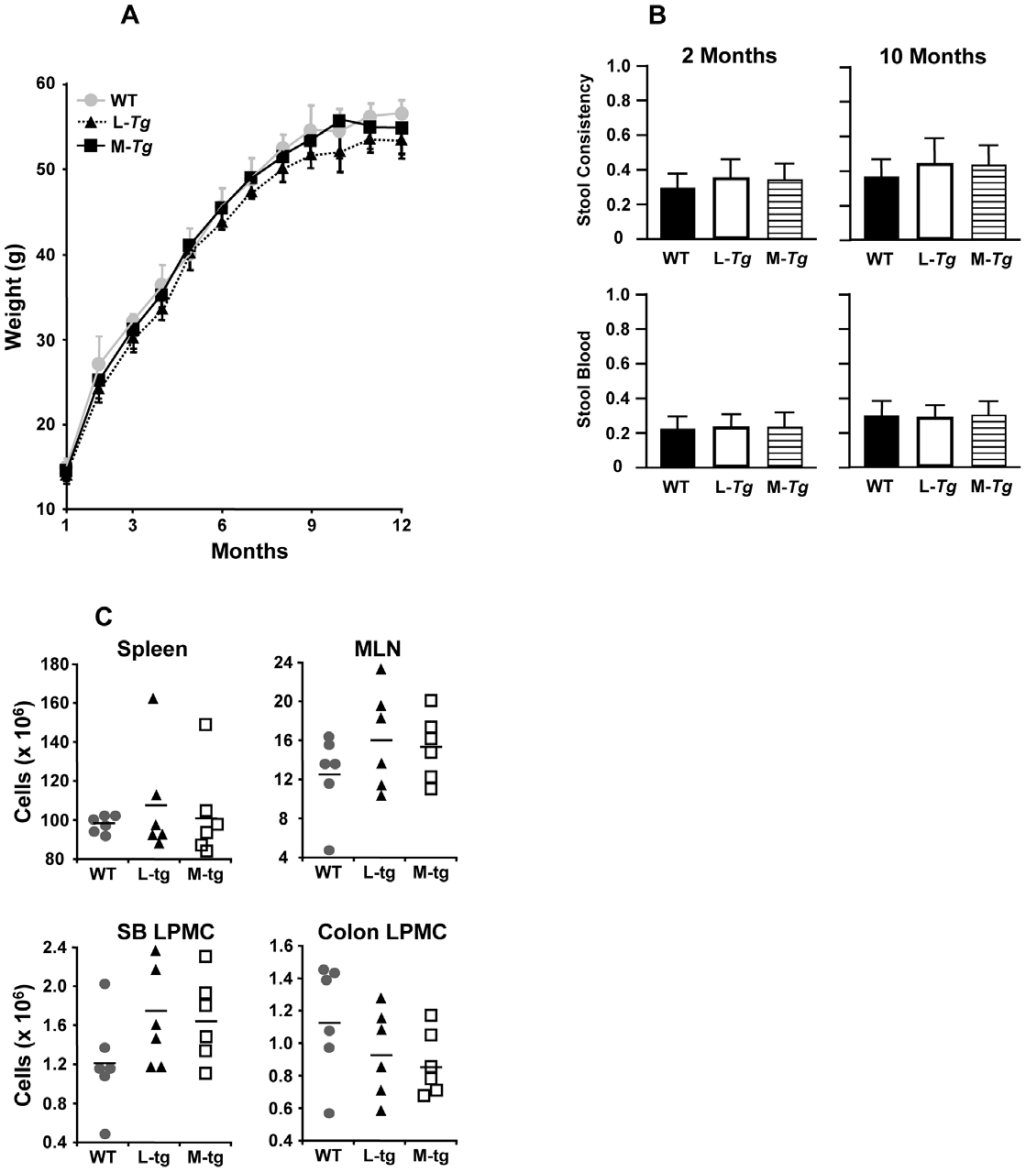
Phenotypic characterization of *Tl1a Tg* mice. (A) Body weights for WT (grey circle), *LCK-CD2-Tl1a Tg* mice (L-*Tg*, black filled triangle) or *FMS-Tl1a Tg* mice (M-*Tg*, black filled square) are shown n = 20 per group. Data are expressed as mean weight in grams (g) ± SD. (B) Stool consistency (top panels) and fecal blood (bottom panels) were determined using standard methods from WT, *LCK-CD2-Tl1a Tg* (L-*Tg*) or *FMS-Tl1a Tg* mice (M-*Tg*) mice [20]. Data are expressed as mean ± SD. N = 20 per group. (C) Total number of cells were isolated from spleen, MLN and lamina propria mononuclear cells (LPMC) from the colon and distal 10 cm of small intestine isolated from 10 months old WT (grey circle), *LCK-CD2-Tl1a Tg* (L-*Tg*, black triangle) or FMS*-Tl1a Tg* mice (M-*Tg*, open square) and represented as absolute cell number x 10^6^. Each data point represents an independent mouse.

Both *FMS-Tl1a-GFP* and *LCK-CD2-Tl1a-GFP Tg* mice are fertile, but are born at less than Mendelian frequency. Mendelian expectation is 50% transmission of the transgene. When *Tg* hemizygous mice are mated to WT mice, the frequency of *FMS-Tl1a-GFP Tg* mice born is 42% (58/138) and in *LCK-CD2-Tl1aTg* mice, the frequency is 40% (51/126). Both the *FMS* and *LCK Tl1a Tg* mice appeared healthy and gained weight at similar rates (Fig. 2A). There were no differences in the disease activity index (DAI) [20] between *Tg* and WT for up to 10 months (Fig. 2B). We also did not observe differences in the splenic cell number, MLN cell number, lamina propria mononuclear cell (LPMC) number in the small bowel or colon, colon length, small bowel (SB) length or spleen size between WT and *Tl1a Tg* mice at 2 or 10 months (Fig. 2C and data not shown). There was a trend toward higher cell numbers in the MLN and SB LPMC in *Tl1a Tg* mice but they did not reach statistical significance (Fig. 2B and data not shown).

### Tl1a expression in APC and T cells induce mild histologic small bowel inflammation

**Figure 3:**
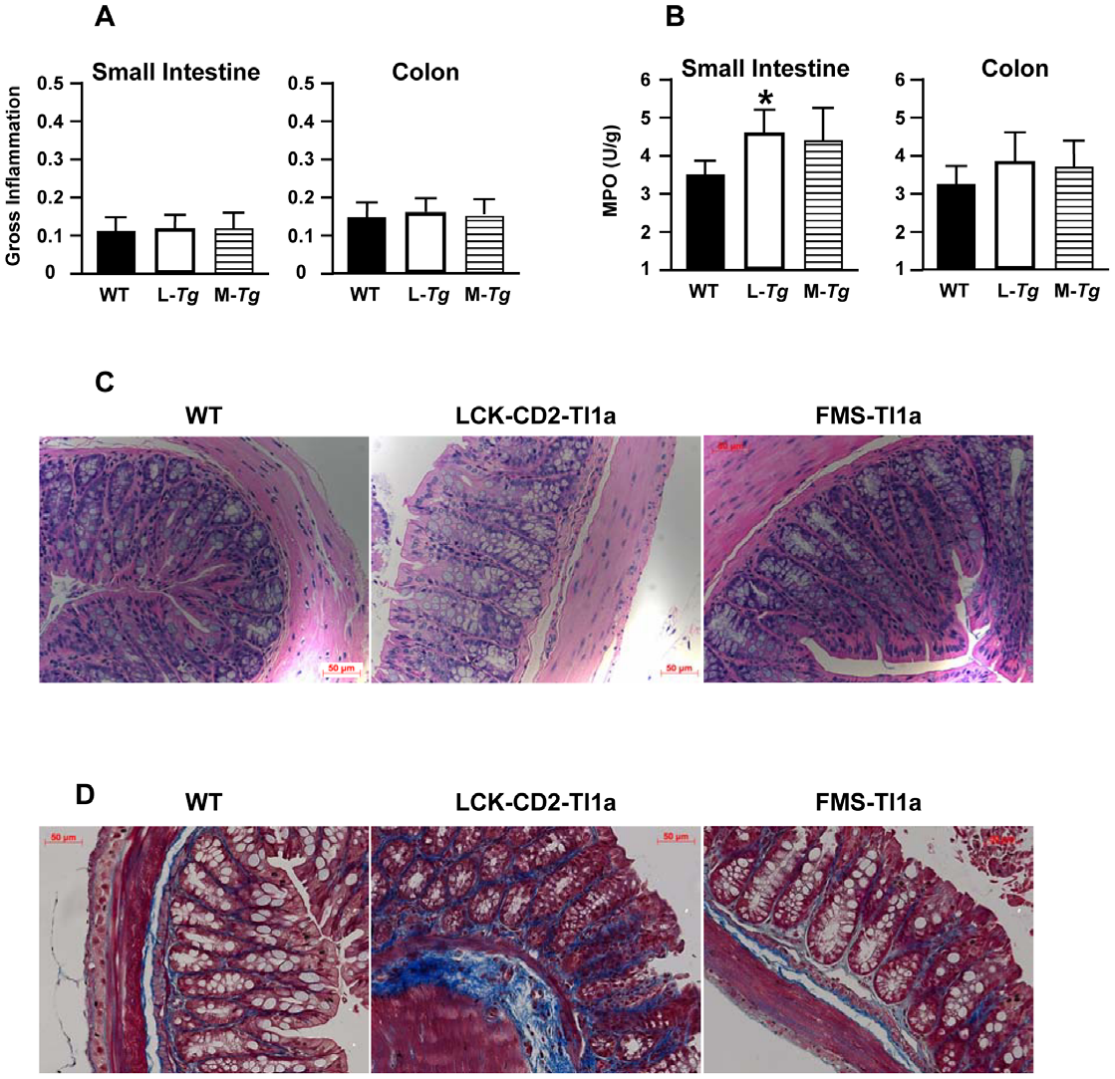
*Tl1a Tg* mice do not develop gross intestinal inflammation but exhibit enhanced colonic fibrosis. (A) Gross appearance (wall thickening, hyperemia, rigidity or adhesions) of small intestine and colon are measured from 10 months old WT, *LCK-CD2-Tl1a Tg* (L-*Tg*) or *FMS-Tl1a Tg* (M-*Tg*) mice using a standard scoring system [21]. Data are expressed as mean ± SD. (B) Myeloperoxidase (MPO) activity is measured on the distal 3 cm of ilea and mid-colon and data are expressed as arbitrary unit (U) per gram (g) of protein. *P<0.05 (C) Representative hematoxylin and eosin (H&E) stained colon section obtained from mid-colon of 10 months old WT, *LCK-CD2-Tl1a Tg* or *FMS-Tl1a Tg* mice are shown. (D) Masson Trichrome staining of collagen deposition in tissue sections of mouse mid-colon. Collagen is stained blue versus red background. There are increased blue collagen stain in *LCK-CD2-Tl1a Tg* and *FMS-Tl1a Tg* compared to WT littermate mice. Magnification 200X. Results are representative of six mice per group for A–D.

Elevated Tl1a expression is implicated in gut mucosal inflammation. We therefore investigated whether mice with elevated Tl1a expression develop spontaneous colitis at 2 months and 10 months of age. The colon and small intestine did not show gross inflammation between WT and *Tg* mice using a standard macroscopic scoring system (Fig. 3A) [21]. Another measure for gut inflammation is to determine myeloperoxidase (MPO) activity [22]. We found significantly increased MPO activity in the small intestine of *LCK-CD2-Tl1a Tg* mice than WT mice (Fig. 3B). MPO activity was similar in the colon of WT and *Tl1a Tg* mice (Fig. 3B). Histological examination of the colon did not reveal increased inflammatory infiltrates, mucin depletion, epithelial cell hyperplasia, abnormal crypt artchitecture, crypt abscess or erosions in either WT or *Tg* mice at 2 or 10 months of age (Fig. 3C and data not shown). As elevated TL1A production in IBD patients is associated with fibrostenotic disease [23], [24], we assessed whether the *Tl1a Tg* mice had increased histologic fibrosis. Notably, we observed increased fibrosis in the colonic mucosa and submucosa of both *LCK-CD2*- and *FMS-Tl1a Tg* mice as compared to WT littermate mice by 10 months of age using the Masson Trichrome stain (Fig. 3D).

**Figure 4:**
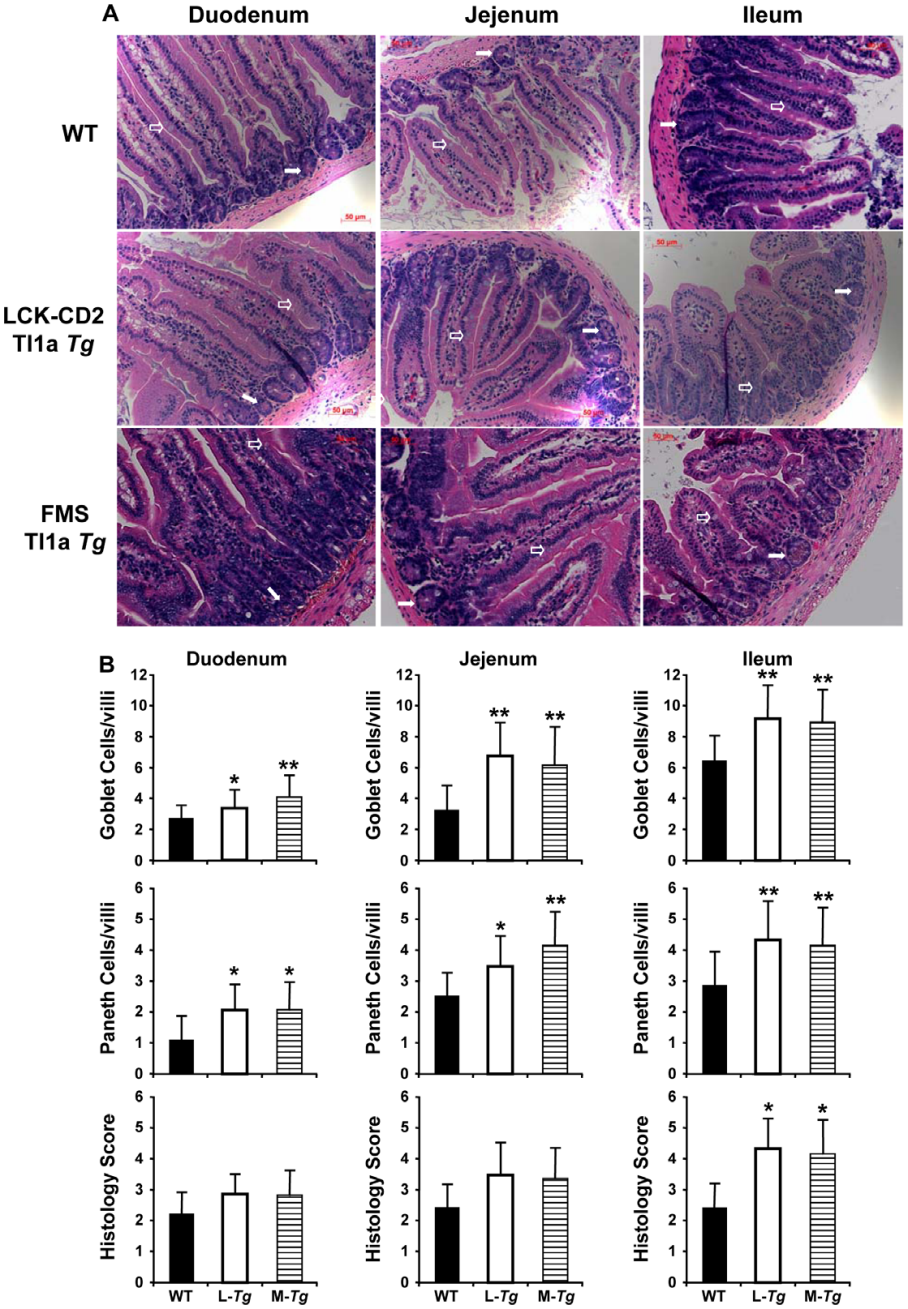
Constitutive Tl1a expression leads to increased numbers of goblet and Paneth cells in the small intestine and ileal histological inflammation. (A) Representative H&E stained sections obtained from the indicated portions of small intestine from 2 months old WT, *LCK-CD2-Tl1a Tg* (L-*Tg*) or *FMS-Tl1a Tg* (M-*Tg*) mice are shown. Goblet cells are denoted by an open arrow. Paneth cells are denoted by a filled arrow. Results are representative of six mice per group. Magnification 200X. (B) The numbers of goblet (top panel) and Paneth cells (middle panel) were determined by examining at least 80 individual villi at the indicated portions of the small intestine from six mice (2 months old) per group by 2 observers blinded to mouse genotype. Data are expressed as mean (SD. Histologic scores (bottom panel) were determined by 2 observers blinded to mice using standard methods [21]. Data are expressed as mean (SD. At least 36 fields from 6 mice per group at 200x magnification were scored. *P<0.05, **P<0.01.

At 2 months of age, histological examination of the small intestine revealed a significant increase in the number of goblet cells and Paneth cells (Fig. 4A and 4B). There was blunting of the villi and increased LPMC in the ileum of both the *LCK-CD2-* and *FMS-Tl1a Tg* mice compared to WT mice (Fig. 4A). These histological changes were reflected by a significant increase in the inflammatory score in the ileum of *Tl1a Tg* mice using a standard quantitative scoring system (Fig. 4B) [21]. We did not observe villus blunting nor increased mononuclear cells in the LP of the duodenum and jejunum between WT and *Tl1a Tg* mice at 2 months of age (Fig. 4A).

**Figure 5:**
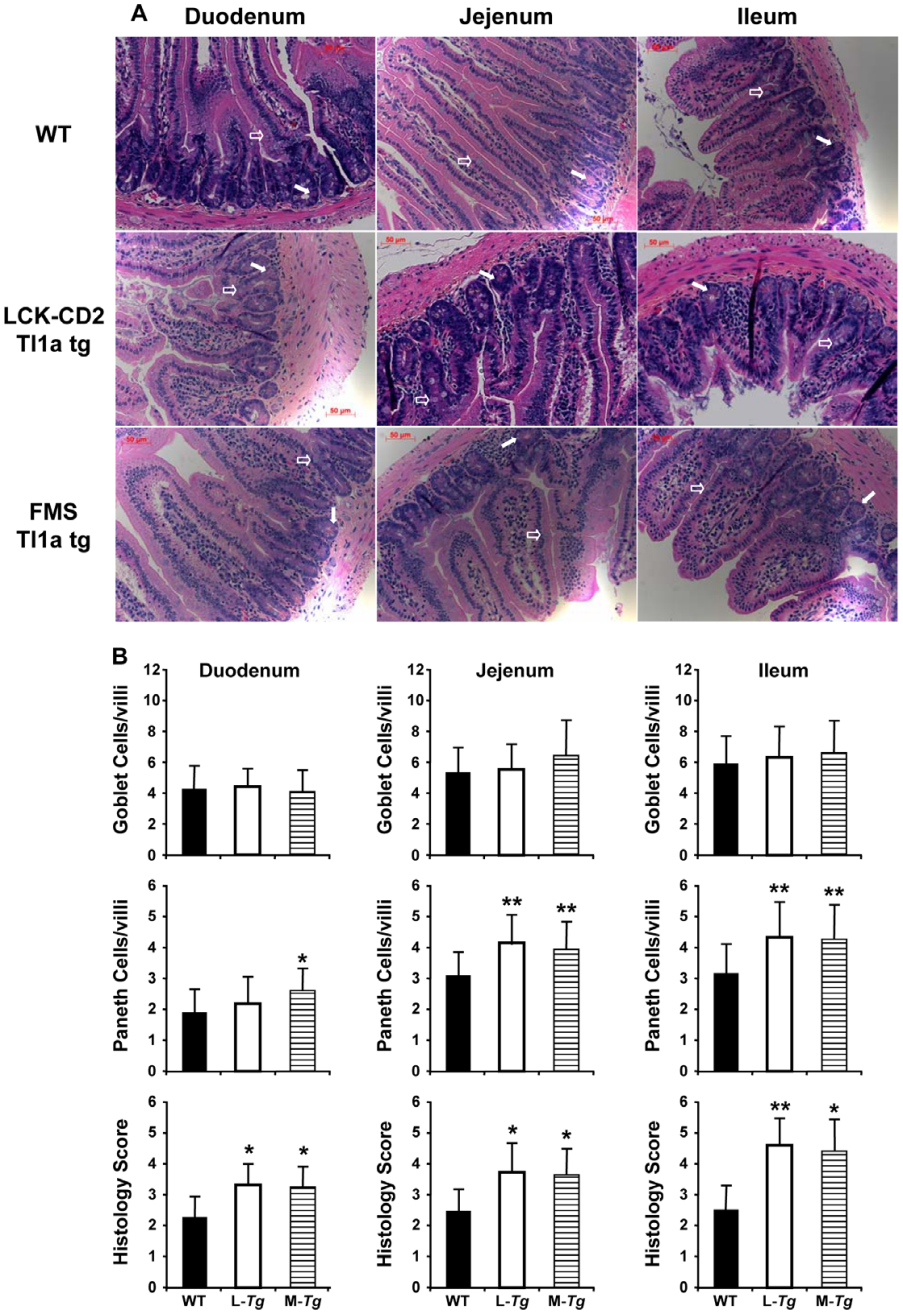
Persistent Paneth cell hyperplasia and worsened small intestinal inflammation as the *Tl1a Tg* mice aged. (A) Representative H&E stained section obtained from the indicated portions of small intestine from 10 months old WT, *LCK-CD2-Tl1a Tg (L-Tg)* or *FMS-Tl1a Tg (M-Tg)* mice are shown. Goblet cells are denoted by an open arrow. Paneth cells are denoted by a filled arrow. Results are representative of six mice per group. Magnification 200X. (B) The numbers of goblet (top panel) and Paneth cells (middle panel) were determined by 2 observers blinded to mice genotype. Histologic scores (bottom panel) were determined from 10 months old WT, *LCK-CD2-Tl1a Tg (L-Tg)*, or *FMS-Tl1a Tg (M-Tg)* mice using standard methods [21]. Quantification of goblet and Paneth cells was determined by examining at least 80 individual villi and histological scores were determined by examining at least 36 fields at 200X magnification. Six independent mice per group were used (A–B). Data are expressed as mean (SD. *P<0.05, **P<0.01.

**Figure 6:**
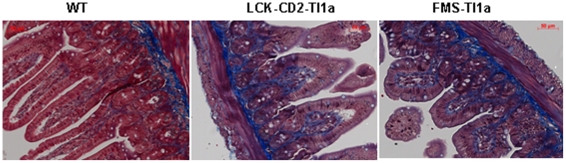
Increased fibrosis in the small intestine of *Tl1a Tg* mice. Masson Trichrome staining was performed on mice ileal sections (distal 3 cm of small intestine). There is increased blue collagen stain in *LCK-CD2-Tl1a Tg* and *FMS-Tl1a Tg* compared to WT littermate mice. Magnification 200X. Six independent mice per group were used.

At 10 months, we similarly observed Paneth cell hyperplasia in both *LCK-CD2-* and *FMS-Tl1a Tg* compared to WT mice (Fig. 5A and 5B). In contrast to younger mice (2 months of age), there were no detectable differences in the number of goblet cells between WT and *Tg* mice at 10 months of age (Fig. 5A and 5B). The inflammatory changes in the small intestine such as increased mononuclear cell infiltrate of the LP and blunting of the villi was more prominent and progressive, involving the duodenum, jejunum and ileum (Fig. 5A and 5B). The increased inflammation in both *LCK-CD2-* and *FMS-Tl1a Tg* mice was associated with increased histologic fibrosis by the more extensive Masson Trichrome stain in the small intestine (Fig. 6). Together, these results indicated that constitutive expression of Tl1a in T cells and myeloid cells lead to progressive spontaneous intestinal inflammation and fibrosis.

### Mice with constitutive Tl1a expression develop extra-intestinal pathology

**Figure 7:**
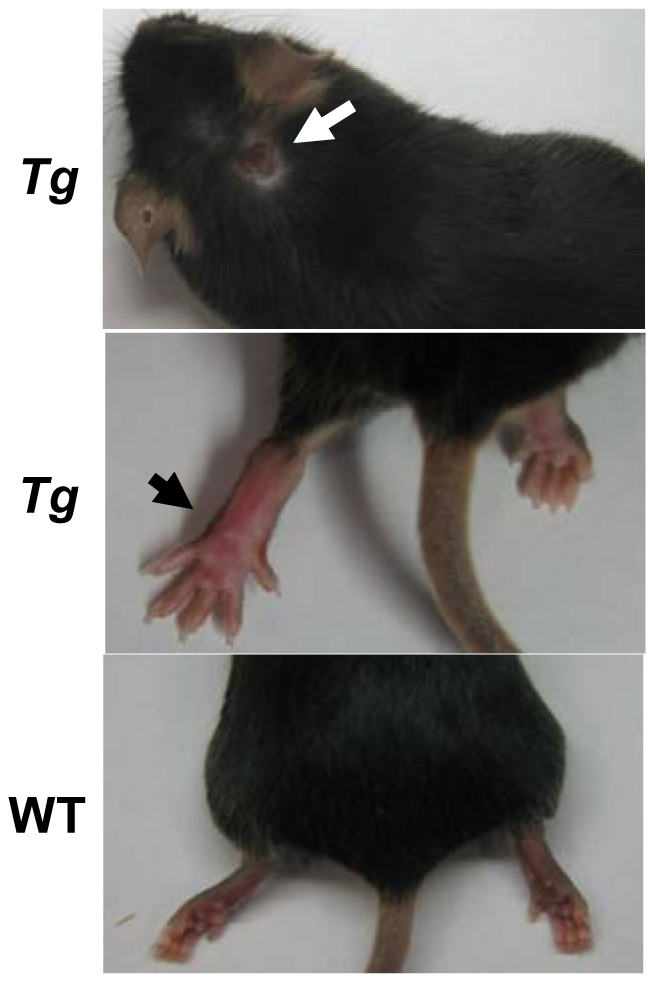
Tl1a transgenic mice develop ulcerated skin lesions and arthropathy. A typical ulcerated skin lesion is illustrated (white arrow, top). Erythematous arthropathy found in the transgenic (*Tg*) mice is shown (black arrow, middle). A WT joint is shown in the bottom panel for comparison.

At a low frequency, we observed extra-intestinal pathology in both the *FMS-Tl1a-* and *LCK-CD2-Tl1a Tg* mice. One such feature was an erythematous ulcerated skin lesion that was observed in 1 out of 58 *FMS-Tl1a Tg*, 2 of 51 *LCK-CD2-Tl1a Tg* and 1 out of 155 WT littermate mice (Fig. 7). Another observed pathology was joint erythema and swelling (Fig. 7) that caused movement difficulties and resultd in mice not able to feed. Arthropathy was observed in 2 out of 58 *FMS Tg*, 2 out of 51 *LCK-CD2 Tl1a Tg* mice and 0 out of 155 WT mice. Three out of 4 *Tg* mice with arthropathy had monoarticular and 1 *LCK*-*CD2-Tl1a Tg* mice had polyarticular disease. Our data suggested these novel murine models with constitutive expression of Tl1a in APC and T cells develop extra-intestinal pathology such as ulcerated skin lesion and arthropathy.

### Accelerated T- and antigen presenting cell activation in LCK-CD2- and FMS-Tl1a *Tg* mice

**Figure 8:**
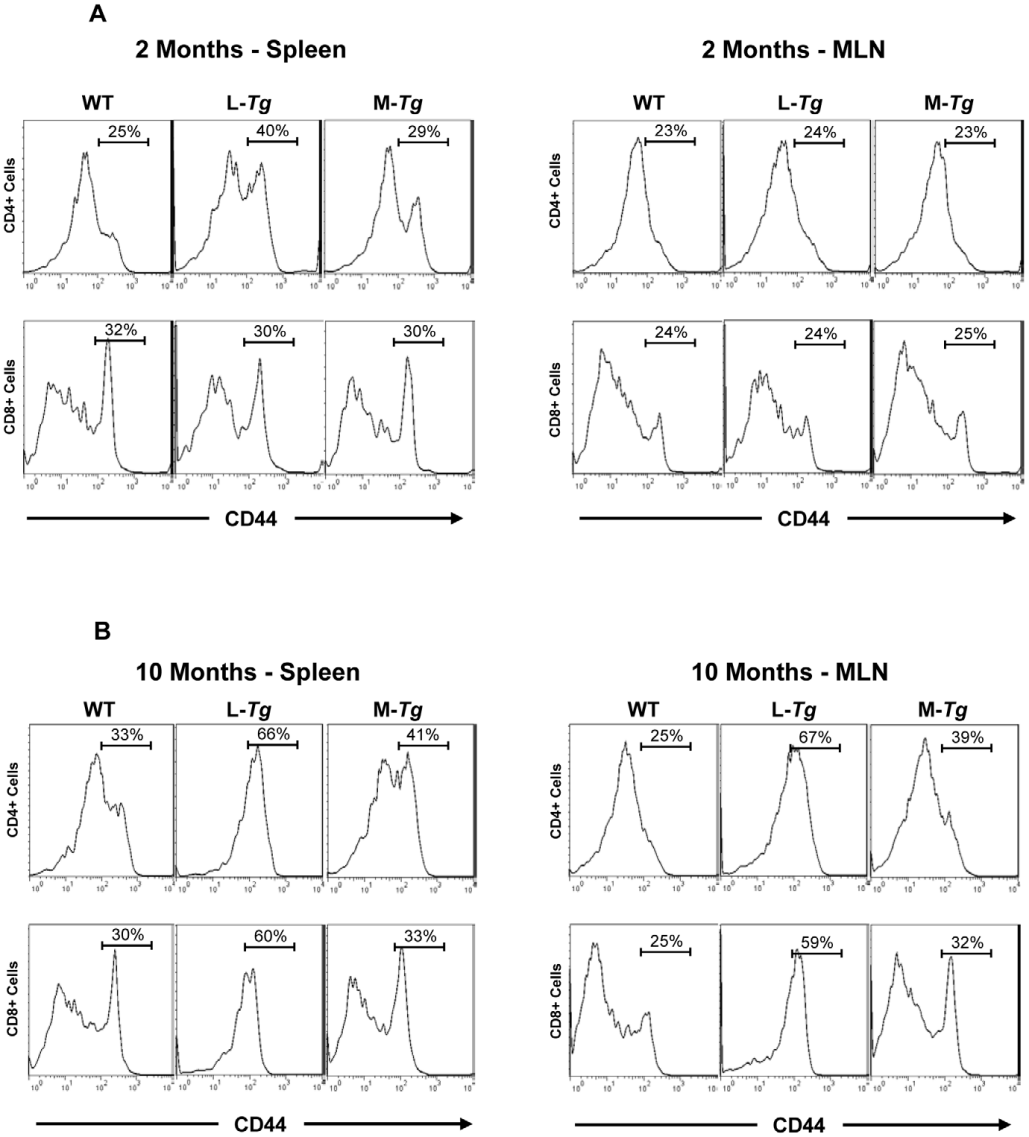
Sustained Tl1a expression leads to an increased percentage of activated T cells. FACS plot of 2 month (A) and 10 month (B) splenocytes and MLN cells showing expression of activation markers CD44. Either CD4^+^ or CD8^+^ cells are gated as indicated. Data shown are representative of 4 mice per group. WT  =  wildtype, L-*Tg*  =  *LCK-CD2-Tl1a-GFP Tg* mice, M-*Tg*  =  *FMS-Tl1a Tg* mice.

To assess whether constitutively expressed Tl1a can co-stimulate T cells *in vivo*, we compared the expression of an activation marker on CD4^+^ and CD8^+^ cells between *Tg* and WT littermate controls. CD4^+^CD45RB^low^CD25^+^ Treg cells were gated out in order to examine the expression of activation markers on conventional T cells. At 2 months, there were almost 2 fold higher CD4^+^CD44^+^ T cells from the spleen but not MLN of *LCK-CD2-Tl1a Tg* mice (Fig. 8A). There was also no difference in the expression of the activation marker CD44 on CD8^+^ T cells in the MLN and spleen at 2 months of age between *Tl1a Tg* mice and WT littermates (Fig. 8A). By 10 months of age, a higher percentage of *Tl1a Tg* CD4^+^ cells in both the spleen and MLN expressed the activation marker CD44, particularly in the *LCK-CD2-Tl1a Tg* mice (Fig. 8B). In contrast, only CD8^+^ cells from the *LCK-CD2-Tl1a Tg* (not *FMS-Tl1a Tg*) spleen and MLN exhibited an increased expression of the activation marker CD44 (Fig. 8B).

**Figure 9:**
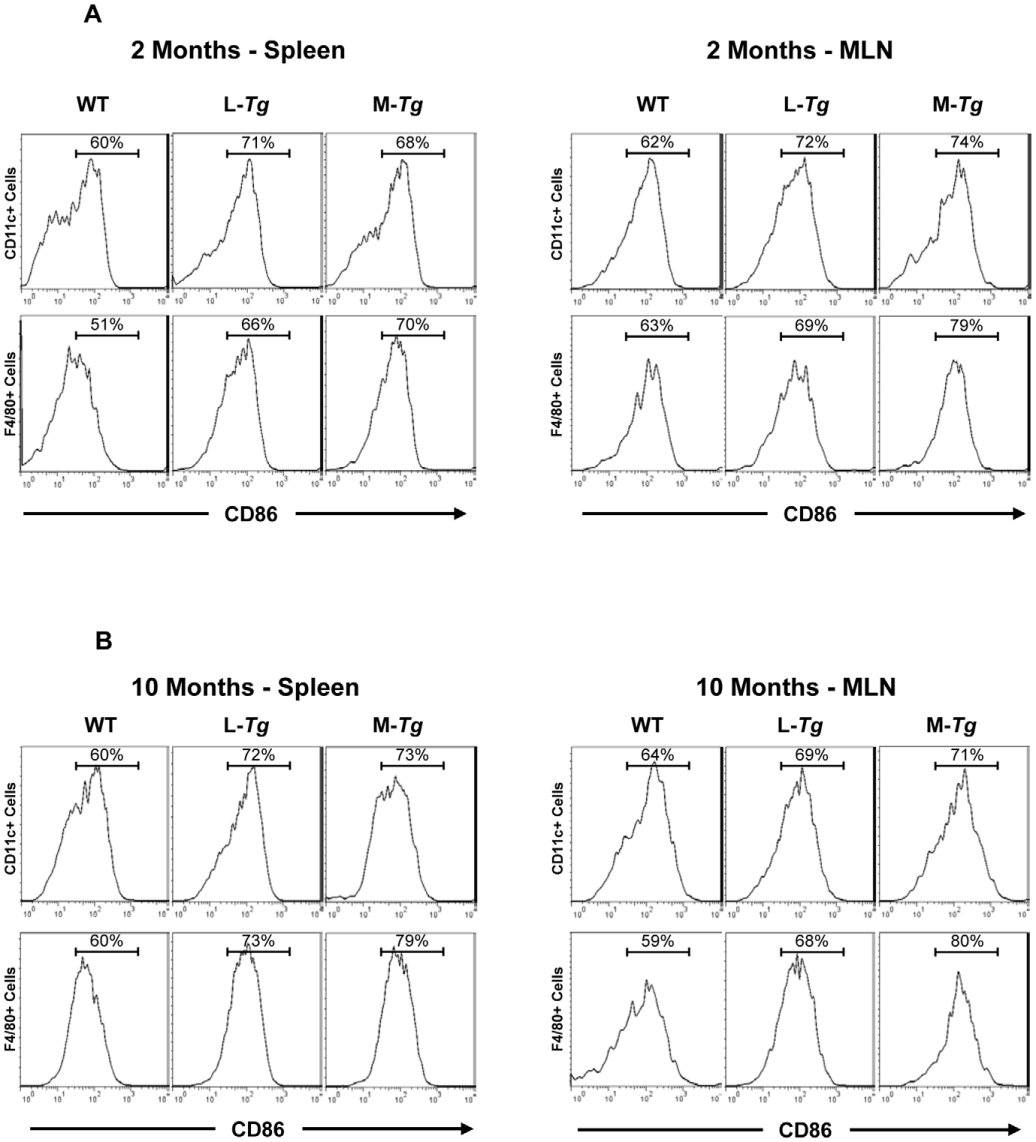
Sustained Tl1a expression leads to an increased percentage of activated DC and macrophages. FACS plot of 2 month (A) and 10 month (B) splenocytes and MLN cells showing expression of activation markers CD86. Either F4/80^+^ or CD11c^+^ cells were gated as indicated. Data shown are representative of 4 mice per group. WT  =  wildtype, L-*Tg*  =  *LCK-CD2-Tl1a-GFP Tg* mice, M-*Tg*  =  *FMS-Tl1a Tg* mice.

To assess the effect of constitutive Tl1a expression on the activation state of DC and macrophages, the expression of the activation marker CD86 was compared between *Tl1a Tg* and WT littermate controls. Flow cytometric (FACS) analysis revealed increased CD86^+^ expression on DC (CD11c+) and macrophages (F4/80+) in both the spleen and MLN of Tl1a *Tg* mice compared to WT mice (Fig. 9A). In contrast to *LCK-CD2-Tl1a Tg* mice, there was a negligible further increase in the percentage of activated DC and macrophages over time (compare 2 and 10 months, Fig. 9A and B). These data demonstrated that sustained Tl1a expression could result in enhanced activation of CD4^+^, CD8^+^, DC and macrophages *in vivo*.

### Constitutive *in vivo* Tl1a expression leads to an increased number of T cells expressing Treg and gut homing markers

**Figure 10:**
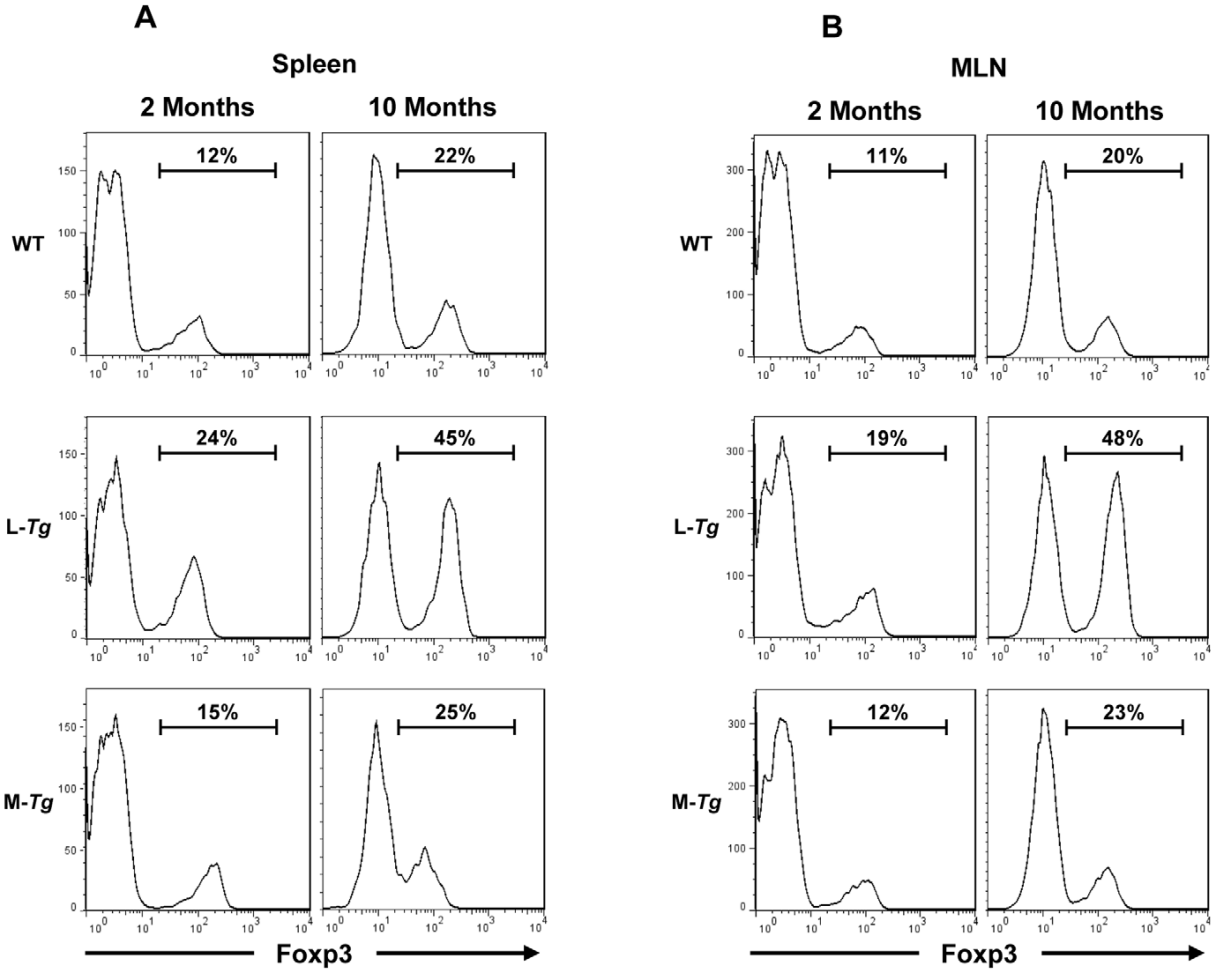
Increased numbers of regulatory T (Treg) cells in *Tl1a Tg* mice. FACS plot of CD4^ +^ Foxp3^+^ splenocytes (A) or CD4^ +^ Foxp3^+^ MLN cells (B) are shown. Data shown are representative of 4 mice per group at either 2 or 10 months of age. WT  =  wildtype, L-*Tg*  =  *LCK-CD2-Tl1a-GFP Tg* mice, M-*Tg*  =  *FMS-Tl1a-GFP Tg* mice.

FACS analysis did not reveal any differences in the frequencies of CD3^+^, CD4^+^, CD8^+^, MHCII^+^, CD11c^+^ or F4/80^+^ cells in the spleen and MLN between *FMS-Tl1a Tg*, *LCK-CD2-Tl1a Tg* or WT littermate mice (data not shown). There was an increase in the frequency of Foxp3 positive cells in the spleen and MLN of *LCK-CD2-Tl1a Tg* mice (Fig. 10A and B). The percentage of Foxp3^+^ cells further increased as the *LCK-CD2-Tl1a Tg* mice aged (Fig. 10A and B). In contrast, there was no difference in the frequency of Foxp3^+^ cells in the spleen or MLN of *FMS-Tl1a Tg* mice at both 2 and 10 months (Fig. 10A and B).

**Figure 11:**
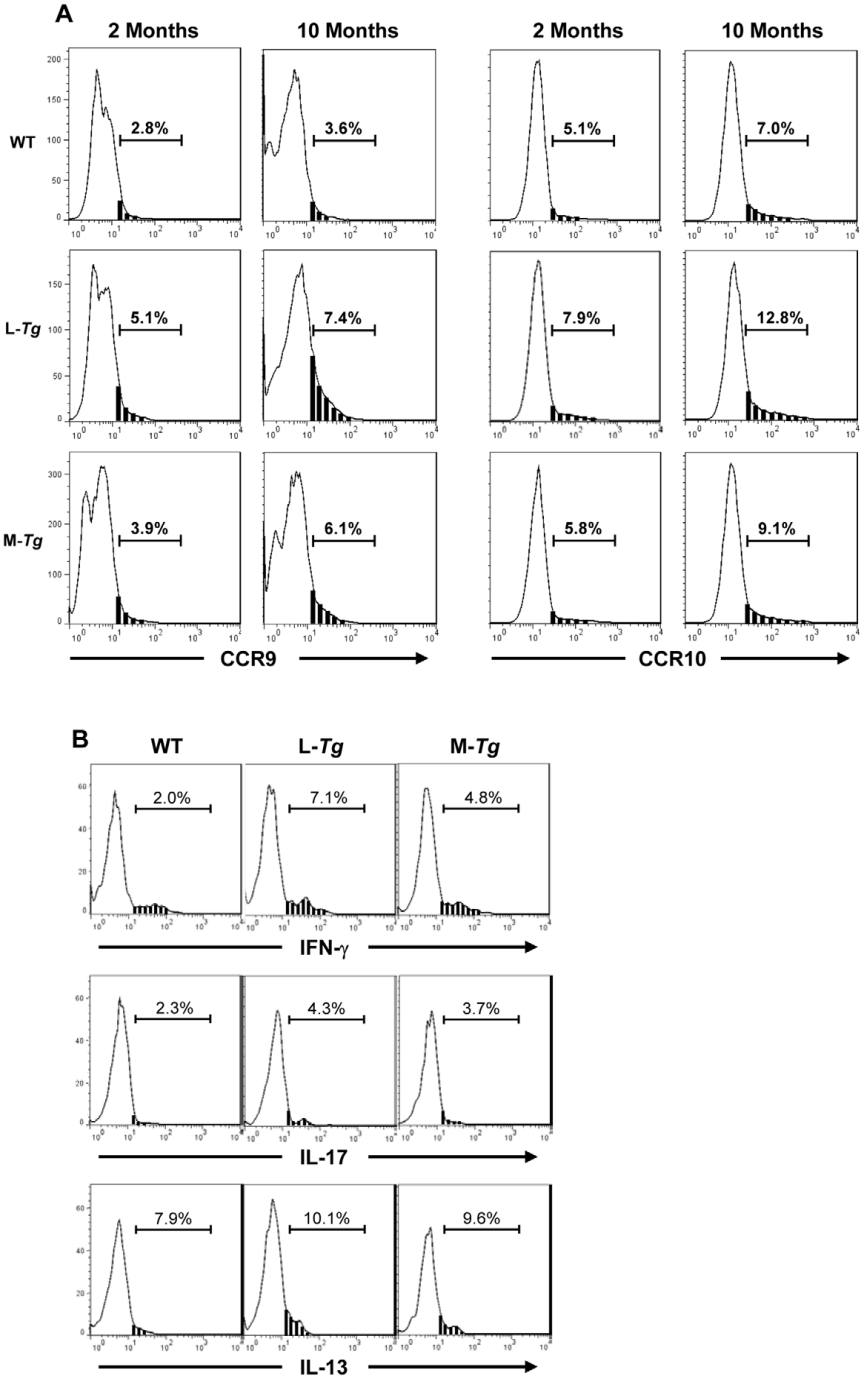
Increased expression of gut homing markers and IFN-γ in *Tl1a Tg* mice. (A) FACS plots showing expression of gut homing markers CCR9 and CCR10 on CD4^+^ cells isolated from the MLN. (B) FACS plot of gated CD4^+^ cells from MLN and stained for intracellular IFN-γ, IL-17, and IL-13 expression from 10 months old WT, *LCK-CD2-Tl1a Tg* (L-*Tg*) or *FMS-Tl1a Tg* (M-*Tg*) mice. Data shown are representative of 4 mice per group (A and B). Black bars indicate area under the curve that is gated as CCR9 positive cells. WT  =  wildtype, L-*Tg*  =  *LCK-CD2-Tl1a-GFP Tg* mice, M-*Tg*  =  *FMS-Tl1a Tg* mice.

A trend toward a higher cell number in the MLN and small bowel LPMC was noted in older (Fig. 2C and 5A). To determine whether this finding was due to increased trafficking to the gut immune compartment, we assessed the expression of the gut homing markers CCR9 and CCR10 in the MLN and spleen of *Tl1a Tg* and WT littermate mice. In the spleen, there was no difference in CCR9^+^ or CCR10^+^ cells at 2 or 10 months between either *Tl1a Tg* or WT mice (data not shown). Notably, we found an increase in the percentage of cells expressing CCR9 and CCR10 in the MLN of *LCK-CD2-Tl1a Tg* mice compared to WT mice at 2 months and the difference became even greater as the mice aged (Fig. 11A). For the *FMS-Tl1a Tg* mice, a higher percentage of CCR9^+^ and CCR10^+^ cells was observed at 10 months (but not at 2 months) (Fig. 11A).

### *Tl1a Tg* mice have an enhanced proinflammatory cytokine profile in the MLN and small intestine

To assess the molecular consequences of increased expression of activation and gut homing marker, we measured the expression of IFN-γ, IL-13 and IL-17 by FACS analysis and ELISA. There was no difference in the expression of CD4^+^IFN-γ^+^, CD4^+^IL-13^+^, CD4^+^IL-17^+^ T cells by either intracellular stain or ELISA at 2 months of age between *FMS-Tl1a Tg*, *LCK-CD2-Tl1a Tg* or WT littermate mice (data not shown). By 10 months of age, the frequency of CD4^+^IFN-γ^+^ and CD4^+^IL-17^+^ T cells increased by approximately 3- and 2- fold respectively, in the MLN of *LCK-CD2-Tl1a Tg* mice (Fig. 11B). In the *FMS-Tl1a Tg* mice, the percentage of CD4^+^IFN-γ^+^ cells increased by approximately 2-fold (Fig. 11B). We did not observe any differences in the CD4^+^IL-13^+^ T cells in both of the *Tl1a Tg* mice and only a negligible difference in the CD4^+^IL-17^+^ T cells in *FMS-Tl1a Tg* mice (Fig. 11B).

**Figure 12:**
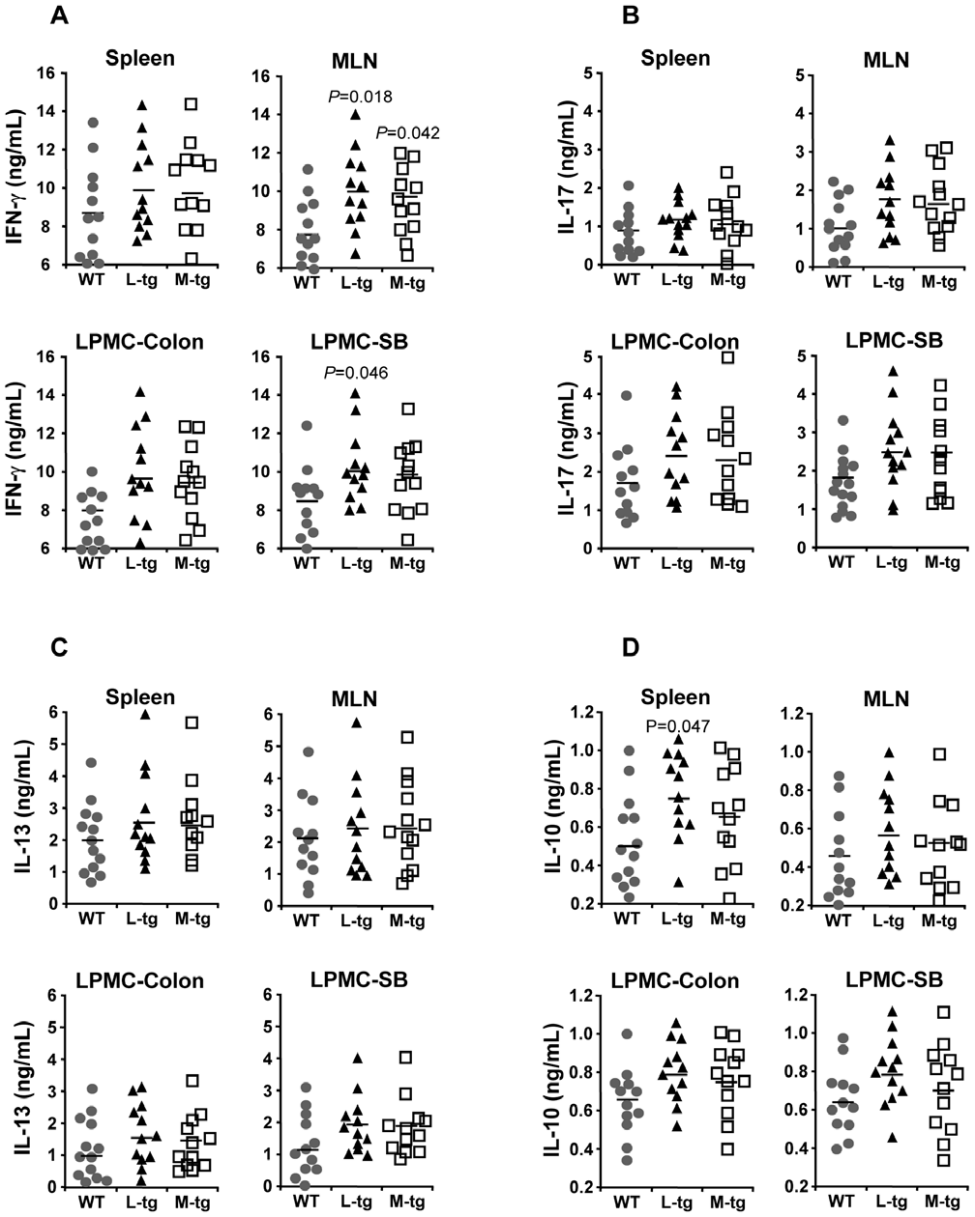
Cytokine profile of *Tl1a Tg* and WT littermate mice. IFN-γ (A), IL-17 (B), IL-13 (C) and IL-10 (D) secretion after stimulation with anti-CD3 and anti-CD28 were assessed by enzyme-linked immunosorbent assay (ELISA). Each data point (A-D) represents cytokine expression for either splenocytes, MLN cells or lamina propria mononuclear cells (LPMC) from either the colon or the distal 10 cm of small intestine isolated from an individual mouse. *p*-values are indicated the figure where significant. WT  =  wildtype, L-*Tg*  =  *LCK-CD2-Tl1a-GFP Tg* mice, M-*Tg*  =  *FMS-Tl1a Tg* mice.

To confirm the FACS findings, we isolated cells from the spleen, MLN and LPMC from the colon and small intestine and assessed their ability to produce cytokines following stimulation with anti-CD3 and anti-CD28. Similar to the intracellular stain, we found significantly higher IFN-γ production in the MLN and small intestine LPMC of *LCK-CD2-Tl1a Tg* mice (Fig. 12A). *FMS-Tl1a Tg* mice also exhibited significantly increased IFN-γ production in MLN and a trend toward a higher level in the LPMC from the small intestine (Fig. 12A). There was also a trend toward higher IL-17 and IL-13 production in the MLN and small intestine LPMC of both *LCK-CD2-Tl1a* and *FMS-Tl1a Tg* mice, but this did not reach statistical significance (Fig. 12B and C). Interestingly, the expression of the anti-inflammatory cytokine IL-10 appeared to be higher in both the *Tl1a Tg* mice and reached statistical significance in the splenic cells of *LCK-CD2-Tl1a Tg* as compared to WT mice (Fig. 12D). These data suggested that constitutive expression of Tl1a *in vivo* resulted in enhanced IFN-γ and IL-10 production and potentially enhanced IL-17 and IL-13 production.

## Discussion

This study shows that constitutive expression of Tl1a on either T- or APC cells leads to progressive histological inflammation in the small intestine and worsened fibrosis in both the small intestine and colon over time. Even though sustained expression of Tl1a in T- or APC did not result in gross macroscopic gut inflammation or colitis symptoms as measured by DAI (weight loss, fecal occult blood or loose stool) for up to 1 year, there were histologically determined inflammation as evidenced by blunting of the small intestinal villi, hyperplasia of goblet and Paneth cells and increased LPMC infiltrate. These histological changes are markers of early gut inflammation that are also found in other murine models of colitis and ileitis [25], [26]. As the small bowel inflammation worsened with age, there was no difference in the goblet cell number between *Tl1a Tg* and WT littermate mice (Fig. 4 and 5). This was likely due to the fact that goblet cell number decreased with worsened inflammation in older *Tl1a Tg* mice [26], [27], [28]. This histologic inflammation was only detected in the small intestine (not colon) and was particularly evident in the ileum. The degree of inflammation in the small intestine was similar between *Tg* mice with sustained Tl1a expression in T- and myeloid cells (Fig. 4).

Another histological finding was increased small intestinal and colonic fibrosis in *Tl1a Tg* compared to WT littermate mice by 10 months of age (Fig. 3D and 6). This could be due to the fact that there was higher transgene expression in the *LCK-CD2-Tl1a Tg* mouse (approximately 90% of T cells) compared to its expression in APC (approximately 70% of DC and macrophages). Alternatively, constitutive Tl1a expression in lymphocytes could be more fibrogenic than its expression on either DC or macrophages. The presence of colonic fibrosis in the absence of detectable histologic inflammation suggested that Tl1a may be a pro-fibrogenic factor in addition to its role in inflammation. Notably, *TL1A haplotype B* which is associated with increased secretion of soluble and membrane TL1A is also characterized by increased need for surgery especially in Jewish patients [23], [24]. Fibrostenotic disease was also more common in this group. It would be medically useful to determine whether downregulating the TL1A/DR3 pathway can prevent the progression of IBD-associated fibrosis since there is currently no effective medical therapy to treat established fibrostenotic disease in IBD patients. It would be interesting to study the interaction between TL1A and other known pro-fibrogenic factors such as transforming growth factor beta 1 (TGF-β1) and insulin-like growth factor 1 (IGF-1) using the chronic 2,4,6-trinitrobenzensulfonic acid (TNBS) colonic injury model which causes both colitis and intestinal fibrosis [29], [30], [31].

We observed a low frequency of extra-intestinal pathology in both *LCK-CD2-Tl1a Tg* and *FMS-Tl1a Tg* mice. These included ulcerated skin lesions and erythematous swollen joints (fig. 7), which are similar to known extra-intestinal manifestations associated with human IBD. The cause of the extra-intestinal pathology in *Tl1a Tg* mice remains to be investigated, but may be related to the differential expression of CCR10 homing molecules in the *Tl1a Tg* mice. In addition to its function as a gut mucosal homing receptor [32], CCR10 is important in lymphocyte trafficking to the skin [33], [34].

The chemokine receptors CCR9 and CCR10 play a major role in *in vivo* lymphocyte trafficking to portals of microbial entry, such as gut mucosal tissues and the skin [33], [34], [35], [36], [37]. The higher LPMC infiltrate in the small intestine as compared to the colon in *Tl1a Tg* mice may be due to the fact that CCR9 is preferentially found in the small intestine [38] and its ligand CCL25/TECK is expressed in both lamina propria venules and small intestine enterocytes [39], [40]. In contrast, colonic expression of CCR9 and its ligand CCL25/TECK is limited [39], [40]. The clinical importance of dysregulated immune trafficking in IBD is highlighted by the efficacious therapies such as natalizumab that blocks gut homing in inducing and maintaining remission in CD [41].

We found that constitutive expression of Tl1a resulted in a progressive activated phenotype over time in CD4^+^ and CD8^+^ T cells (Fig. 8), macrophages and DC (Fig. 9). Increased T cell and APC activation in *Tl1a Tg* mice may be a direct consequence of constitutive TL1a costimulation of DR3 expressing T cells and macrophages [11], [42], [43] or indirectly through failure of immunological tolerance leading to enhanced activation. In fact, recent studies have shown that the TL1A-DR3 pathway can attenuate the suppressive effect of Tregs on effector T cells [16], [17]. The activated phenotype of T and APC in combination with the constitutive costimulation of TL1A-DR3 signaling pathway may lead to the significantly higher IFN-γ expression (Fig. 11B and 12A) that we saw in the *Tl1a Tg* mice, which is consistent with the previous observation that TL1A is involved in mediating the Th1 response [4], [5], [8], [9], [13], [14], [15], [44], [45]. There was also a trend toward increased IL-13 and IL-17 expression in mice with constitutive Tl1a expression (Fig. 12B and C), which was consistent with the role of TL1A in Th2- and Th17- mediated functions in various mouse models [9], [10], [11], [12], [16], [17]. Lastly, we found significantly increased IL-10 production in *LCK-CD2-Tl1a Tg* mice (Fig. 12D), which could be related to higher percentage of Foxp3^+^ Treg cells in *LCK-CD2-Tl1a Tg* as compared to *FMS-Tl1a Tg* or WT mice (Fig. 10A and B).

Recently, two groups independently reported the generation of transgenic mice with sustained TL1A expression in DC and T cells. Our results were similar to the constitutive Tl1a expressing *Tg* mice in Meylan et al. and Taraban et al., with the increased goblet, Paneth and Treg cells, ileitis and activated phenotype in T and APC cells [16], [17]. However, a major difference was that the ileitis found in the recently published *Tl1a Tg mice* appeared to be mainly attributed to Th2/IL-13 effector pathway [16], [17], whereas we observed a higher production of IFN-γ from the *Tl1a Tg* mice generated in this study (Fig. 11B and 12A). In addition, we observed extra-intestinal pathology and increased gut fibrosis in the *Tl1a Tg* mice, which were not reported in the other *Tl1a Tg* mice. Several reasons may account for the phenotypical differences between the *Tl1a Tg* mice. One explanation may be methodological differences. For example, different promoter/enhancer elements were used to drive tissue specific expression of the different *Tl1a Tg* mice. We used the *LCK-CD2* promoter/enhancer element [19], whereas the CD2 promoter [46] was used to generate the T cell specific *Tl1a Tg* mice [16]. The *c-fms* promoter vector [18] was used to generate myeloid specific *Tl1a Tg* mice for this report whereas the *CD11c* promoter [47] was used for the recently published studies [16], [17]. Differences in the phenotype of the transgenic mice using the different T cell promoter/enhancer element to drive transgene expression was observed previously in the T cell specific *LIGHT Tg* mice [19], [48], [49]. Another reason may lie in the mouse strain used. The *Tl1a Tg* mice used in this report were generated by direct microinjection into C57BL/6 pronuclei whereas the DC specific *Tl1a Tg* mouse [17] was generated by microinjecting into FVB/N zygotes and subsequently backcrossed 4–5 generations to the C57BL/6 strain. Alternatively, these phenotypical differences may be due to the different gut microflora between the animal housing facilities. Previous studies have demonstrated that genetically identical inbred mice strain raised in different facilities had different immune composition due to the different intestinal microbiota composition [50], [51], [52]. The potential immunomodulatory role of gut microbiota in the different *Tl1a Tg* mice highlights the previously described role of the TL1A-DR3 signaling pathway in bacteria recognition and microbial-host interactions [6], [8], [10], [16].

In summary, we generated tissue specific, *in vivo* expression of Tl1a in T cells and APC and found that it lead to patchy small bowel inflammation and fibrosis in both the small intestine and the colon. Sustained expression of Tl1a leads to an activated immune phenotype, increased expression of gut homing molecules and Th1 (and possibly Th17 and Th2) responses. These novel Tl1a transgenic models of intestinal inflammation may be useful to study fibrotic response in inflammation and the pathogenesis of various disorders of immune dysregulation such as IBD.

## Materials and Methods

### Transgenic mice and genotype

Murine *Tl1A* cDNA (Open Biosystems, Clone ID 30740802) was digested by EcoRI/SmaI and inserted into the EcoRI/SmaI site of pIRES2-EGFP (Clontech). To generate lymphoid specific Tl1A transgenic mice, DNA fragment containing murine *Tl1A-IRES2-EGFP* was amplified by PCR using sense primer 5′-AATGGGGGCGCGCCGGGCTCTCTGGTCAGAAGGGATCAG-3′ and antisense primer 5′-TTTACGGGCGCGCCCCTTAAGATACATTGATGAGTTTGG-3′, digested with AscI, and cloned into AscI site of plck.E2 (generous gift from T. Hettmann, The University of Chicago), which contains the proximal *lck* promoter, human growth hormone gene (polyadenylation site), and locus control region elements from the human *CD2* gene to generate plasmid pLCK-Tl1a-IRES2-EGFP. The plck.E2 has been used to mediate T cell lineage specific expression [19]. The *Tl1A-IRES2-EGFP* fragment was sequenced to confirm that no mutations were generated during the cloning process. A 10-kb fragment was excised by NotI digest and used for microinjection into C57BL/6 pronuclei performed by Caliper Life Sciences. 8 independent murine lines containing genomic integration of the *Tl1a transgene* were identified by PCR using the following primers: 5′- GACTAACAAAGATGCCTGCCTGTGG-3′ and 5′-GCCATCCTTCTGCTGTCTTGGAGA-3′. Only 2 of the 8 lines showed T-cell lineage specific expression of TL1A (95% of T cells and 34% of T cells). We used the founder transgenic (*Tg*) mouse line with *Tl1A* transgene expression in 95% of T cells and called it *LCK-CD2-Tl1A-GFP Tg* mouse.

To generate myeloid specific *Tl1a Tg* mice, a XhoI/EcoRI fragment was blunt ended by klenow (NEB) and cloned to blunt ended MluI site of *c-fms* promoter vector (generous gift from D. Underhill, Cedars-Sinai Medical Center). Tl1A-IRES2-EGFP fragment was sequenced to confirm that no mutations were generated during the cloning process. The *c-fms* promoter vector has been used previously to drive expression for cells of the mononuclear phagocyte lineage including macrophages and dendritic cells [18]. A 10-kb fragment was excised by XhoI/ClaI digest and used for microinjection into C57BL/6 pronuclei performed by Caliper Life Sciences. 6 independent murine lines containing genomic integration of the *Tl1a* transgene were identified by PCR using the following primers: 5′-TTGGAAGCTGATTGAAGGGTCCA-3′ and 5′-AGCTCCTCTGCCATCCTTCTGCT-3′. Two of the six lines showed myeloid specific expression of the transgene in approximately 70% of macrophages and dendritic cells, but one has higher GFP level than the other founder line. We used the founder Tg line with the higher GFP expression and called this line *FMS-Tl1a-GFP Tg* mouse.

All mice were maintained under specific pathogen-free conditions in the Animal Care Facility at Cedars-Sinai Medical Center. Littermate control mice were used for all experiments. This study was carried out in strict accordance with the recommendations in the Guide for the Care and Use of Laboratory Animals of the NIH. Mice used in all experiments were specifically approved by Cedars-Sinai Medical Center Animal Care and Use Committee. Approved protocol 2269 was used for this study.

### Disease activity index (DAI), macroscopic and histopathological analysis

Mice were weighed and inspected for diarrhea and rectal bleeding once a week for the first 2 months after weaning, then once a month thereafter. The DAI (combined score of weight loss, presence of blood in stool, and stool consistency) was determined according to a standard scoring system previously described [20]. Colon and small intestine were scored for macroscopic evidence of inflammation using the established classification [21]. Tissue was fixed in 10% neutral buffered formalin (Sigma). Samples were embedded, sectioned, and stained with hematoxylin and eosin by the Histology Core at Cedars-Sinai Medical Center. Masson Trichrome staining was performed as described previously [29]. Histopathological scores of colons and small intestine were assigned in a blinded manner by at least 2 trained pathologists (DQS and HWK) using previously established methods [21]. The histologic score is calculated from observation of at least 36 different fields of stained sections at 200X from 6 mice in each group. The numbers of goblet cells and Paneth cells were determined by examining at least 80 individual villi and crypts from each group.

### Real-time PCR analysis

Total RNA was isolated as previously described [8]. Two micrograms of total RNA was used in each RT reaction using the Omniscript kit (Qiagen), with oligo dT as primer. TL1A and β-actin transcripts were amplified by quantitative real-time RT-PCR as previously described [9]. Duplicates differing by less than one cycle were averaged and amount of transcript was analyzed and expressed as percentage of β-actin.

### Cell isolation and culture

LPMC isolation, culture, and stimulation by anti-CD28 and anti-CD3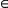 were carried out as previously reported [9]. We used the whole colon for LPMC isolation. We used the distal 10 cm of the SB for LPMC isolation. Cytokine concentration was assayed by enzyme-linked immunosorbent assay (ELISA) using ELISA kits for IFN-γ, IL-17 IL-13, and IL-10 (eBioscience) per manufacture protocol and as previously described [9].

### Flow cytometry and measurement of intracellular cytokine expression

Cells were stained with antibodies against murine CD4 (RM4-5), CD44 (IM7), CD86 (GL1) (BD Biosciences), CCR9 (242503), CCR10 (248918) (R&D systems), CD8 (53-6.7), CD11c (N418), F4/80 (BM8) (Biolegend) and FoxP3 (FJK-165) (eBiosciences). CD16/CD32 (clone 2.4G2) was used to block nonspecific FcR binding (eBiosciences). Intracellular staining was performed with antibodies against IFN-γ (XMG1.2), IL-17 (17B7) and IL13 (13a) (eBioSciences). For determination of intracellular cytokine production by leukocytes, cells were incubated for 5 hours at 37°C with BD Leukocyte Activation Cocktail with BD GolgiPlug (BD Biosciences). For FoxP3 staining, fixation and permeabilization buffers provided from the manufacturer (eBiosciences) were used. Data of the stained cells were acquired on a Cyan flow cytometer (Dako-Cytomation) and analyzed using FlowJo analysis software (Tree Star, Inc).

### Statistical analysis

Data are presented as the mean ± standard deviation. Comparison between two groups was performed by a two tailed, Student's *t*-test. A value of *p*<0.05 was considered significant.

## References

[1] Shih DQ, Targan SR (2009). Insights into IBD Pathogenesis.. Curr Gastroenterol Rep.

[2] Shih DQ, Targan SR, McGovern D (2008). Recent advances in IBD pathogenesis: genetics and immunobiology.. Curr Gastroenterol Rep.

[3] Stappenbeck TS, Rioux JD, Mizoguchi A, Saitoh T, Huett A (2010). Crohn disease: A current perspective on genetics, autophagy and immunity.. Autophagy.

[4] Prehn JL, Mehdizadeh S, Landers CJ, Luo X, Cha SC (2004). Potential role for TL1A, the new TNF-family member and potent costimulator of IFN-gamma, in mucosal inflammation.. Clin Immunol.

[5] Papadakis KA, Prehn JL, Landers C, Han Q, Luo X (2004). TL1A synergizes with IL-12 and IL-18 to enhance IFN-gamma production in human T cells and NK cells.. J Immunol.

[6] Cassatella MA, Pereira-da-Silva G, Tinazzi I, Facchetti F, Scapini P (2007). Soluble TNF-like cytokine (TL1A) production by immune complexes stimulated monocytes in rheumatoid arthritis.. J Immunol.

[7] Prehn JL, Thomas LS, Landers CJ, Yu QT, Michelsen KS (2007). The T cell costimulator TL1A is induced by FcgammaR signaling in human monocytes and dendritic cells.. J Immunol.

[8] Shih DQ, Kwan LY, Chavez V, Cohavy O, Gonsky R (2009). Microbial induction of inflammatory bowel disease associated gene TL1A (TNFSF15) in antigen presenting cells.. Eur J Immunol.

[9] Takedatsu H, Michelsen KS, Wei B, Landers CJ, Thomas LS (2008). TL1A (TNFSF15) regulates the development of chronic colitis by modulating both T-helper 1 and T-helper 17 activation.. Gastroenterology.

[10] Meylan F, Davidson TS, Kahle E, Kinder M, Acharya K (2008). The TNF-family receptor DR3 is essential for diverse T cell-mediated inflammatory diseases.. Immunity.

[11] Fang L, Adkins B, Deyev V, Podack ER (2008). Essential role of TNF receptor superfamily 25 (TNFRSF25) in the development of allergic lung inflammation.. J Exp Med.

[12] Pappu BP, Borodovsky A, Zheng TS, Yang X, Wu P (2008). TL1A-DR3 interaction regulates Th17 cell function and Th17-mediated autoimmune disease.. J Exp Med.

[13] Bamias G, Mishina M, Nyce M, Ross WG, Kollias G (2006). Role of TL1A and its receptor DR3 in two models of chronic murine ileitis.. Proc Natl Acad Sci U S A.

[14] Papadakis KA, Zhu D, Prehn JL, Landers C, Avanesyan A (2005). Dominant role for TL1A/DR3 pathway in IL-12 plus IL-18-induced IFN-gamma production by peripheral blood and mucosal CCR9+ T lymphocytes.. J Immunol.

[15] Bamias G, Martin C, Marini M, Hoang S, Mishina M (2003). Expression, localization, and functional activity of TL1A, a novel Th1-polarizing cytokine in inflammatory bowel disease.. J Immunol.

[16] Meylan F, Song YJ, Fuss I, Villarreal S, Kahle E (2010). The TNF-family cytokine TL1A drives IL-13-dependent small intestinal inflammation.. Mucosal Immunol.

[17] Taraban VY, Slebioda TJ, Willoughby JE, Buchan SL, James S (2010). Sustained TL1A expression modulates effector and regulatory T-cell responses and drives intestinal goblet cell hyperplasia.. Mucosal Immunol.

[18] Sasmono RT, Oceandy D, Pollard JW, Tong W, Pavli P (2003). A macrophage colony-stimulating factor receptor-green fluorescent protein transgene is expressed throughout the mononuclear phagocyte system of the mouse.. Blood.

[19] Wang J, Lo JC, Foster A, Yu P, Chen HM (2001). The regulation of T cell homeostasis and autoimmunity by T cell-derived LIGHT.. J Clin Invest.

[20] Rachmilewitz D, Karmeli F, Shteingart S, Lee J, Takabayashi K (2006). Immunostimulatory oligonucleotides inhibit colonic proinflammatory cytokine production in ulcerative colitis.. Inflamm Bowel Dis.

[21] Ostanin DV, Pavlick KP, Bharwani S, D'Souza D, Furr KL (2006). T cell-induced inflammation of the small and large intestine in immunodeficient mice.. Am J Physiol Gastrointest Liver Physiol.

[22] Katakura K, Lee J, Rachmilewitz D, Li G, Eckmann L (2005). Toll-like receptor 9-induced type I IFN protects mice from experimental colitis.. J Clin Invest.

[23] Picornell Y, Mei L, Taylor K, Yang H, Targan SR (2007). TNFSF15 is an ethnic-specific IBD gene.. Inflamm Bowel Dis.

[24] Michelsen KS, Thomas LS, Taylor KD, Yu QT, Mei L (2009). IBD-associated TL1A gene (TNFSF15) haplotypes determine increased expression of TL1A protein.. PLoS One.

[25] Vidrich A, Buzan JM, Barnes S, Reuter BK, Skaar K (2005). Altered epithelial cell lineage allocation and global expansion of the crypt epithelial stem cell population are associated with ileitis in SAMP1/YitFc mice.. Am J Pathol.

[26] Kosiewicz MM, Nast CC, Krishnan A, Rivera-Nieves J, Moskaluk CA (2001). Th1-type responses mediate spontaneous ileitis in a novel murine model of Crohn's disease.. J Clin Invest.

[27] Shinoda M, Shin-Ya M, Naito Y, Kishida T, Ito R (2010). Early-stage blocking of Notch signaling inhibits the depletion of goblet cells in dextran sodium sulfate-induced colitis in mice.. J Gastroenterol.

[28] Kajino-Sakamoto R, Inagaki M, Lippert E, Akira S, Robine S (2008). Enterocyte-derived TAK1 signaling prevents epithelium apoptosis and the development of ileitis and colitis.. J Immunol.

[29] Koon HW, Shih D, Karagiannides I, Zhao D, Fazelbhoy Z (2010). Substance P Modulates Chronic Inflammation-Induced Colonic Fibrosis.. Am J Pathol.

[30] Di Sabatino A, Jackson CL, Pickard KM, Buckley M, Rovedatti L (2009). Transforming growth factor beta signalling and matrix metalloproteinases in the mucosa overlying Crohn's disease strictures.. Gut.

[31] Simmons JG, Pucilowska JB, Keku TO, Lund PK (2002). IGF-I and TGF-beta1 have distinct effects on phenotype and proliferation of intestinal fibroblasts.. Am J Physiol Gastrointest Liver Physiol.

[32] Hieshima K, Kawasaki Y, Hanamoto H, Nakayama T, Nagakubo D (2004). CC chemokine ligands 25 and 28 play essential roles in intestinal extravasation of IgA antibody-secreting cells.. J Immunol.

[33] Reiss Y, Proudfoot AE, Power CA, Campbell JJ, Butcher EC (2001). CC chemokine receptor (CCR)4 and the CCR10 ligand cutaneous T cell-attracting chemokine (CTACK) in lymphocyte trafficking to inflamed skin.. J Exp Med.

[34] Soler D, Humphreys TL, Spinola SM, Campbell JJ (2003). CCR4 versus CCR10 in human cutaneous TH lymphocyte trafficking.. Blood.

[35] Wurbel MA, Malissen M, Guy-Grand D, Meffre E, Nussenzweig MC (2001). Mice lacking the CCR9 CC-chemokine receptor show a mild impairment of early T- and B-cell development and a reduction in T-cell receptor gammadelta(+) gut intraepithelial lymphocytes.. Blood.

[36] Marsal J, Svensson M, Ericsson A, Iranpour AH, Carramolino L (2002). Involvement of CCL25 (TECK) in the generation of the murine small-intestinal CD8alpha alpha+CD3+ intraepithelial lymphocyte compartment.. Eur J Immunol.

[37] Svensson M, Marsal J, Ericsson A, Carramolino L, Broden T (2002). CCL25 mediates the localization of recently activated CD8alphabeta(+) lymphocytes to the small-intestinal mucosa.. J Clin Invest.

[38] Zabel BA, Agace WW, Campbell JJ, Heath HM, Parent D (1999). Human G protein-coupled receptor GPR-9-6/CC chemokine receptor 9 is selectively expressed on intestinal homing T lymphocytes, mucosal lymphocytes, and thymocytes and is required for thymus-expressed chemokine-mediated chemotaxis.. J Exp Med.

[39] Kunkel EJ, Campbell JJ, Haraldsen G, Pan J, Boisvert J (2000). Lymphocyte CC chemokine receptor 9 and epithelial thymus-expressed chemokine (TECK) expression distinguish the small intestinal immune compartment: Epithelial expression of tissue-specific chemokines as an organizing principle in regional immunity.. J Exp Med.

[40] Papadakis KA, Prehn J, Nelson V, Cheng L, Binder SW (2000). The role of thymus-expressed chemokine and its receptor CCR9 on lymphocytes in the regional specialization of the mucosal immune system.. J Immunol.

[41] Targan SR, Feagan BG, Fedorak RN, Lashner BA, Panaccione R (2007). Natalizumab for the treatment of active Crohn's disease: results of the ENCORE Trial.. Gastroenterology.

[42] Kang YJ, Kim WJ, Bae HU, Kim DI, Park YB (2005). Involvement of TL1A and DR3 in induction of pro-inflammatory cytokines and matrix metalloproteinase-9 in atherogenesis.. Cytokine.

[43] McLaren JE, Calder CJ, McSharry BP, Sexton K, Salter RC (2010). The TNF-like protein 1A-death receptor 3 pathway promotes macrophage foam cell formation in vitro.. J Immunol.

[44] Migone TS, Zhang J, Luo X, Zhuang L, Chen C (2002). TL1A is a TNF-like ligand for DR3 and TR6/DcR3 and functions as a T cell costimulator.. Immunity.

[45] Biener-Ramanujan E, Gonsky R, Ko B, Targan SR (2010). Functional signaling of membrane-bound TL1A induces IFN-gamma expression.. FEBS Lett.

[46] Zhumabekov T, Corbella P, Tolaini M, Kioussis D (1995). Improved version of a human CD2 minigene based vector for T cell-specific expression in transgenic mice.. J Immunol Methods.

[47] Brocker T, Riedinger M, Karjalainen K (1997). Targeted expression of major histocompatibility complex (MHC) class II molecules demonstrates that dendritic cells can induce negative but not positive selection of thymocytes in vivo.. J Exp Med.

[48] Shaikh RB, Santee S, Granger SW, Butrovich K, Cheung T (2001). Constitutive expression of LIGHT on T cells leads to lymphocyte activation, inflammation, and tissue destruction.. J Immunol.

[49] Wang J, Anders RA, Wu Q, Peng D, Cho JH (2004). Dysregulated LIGHT expression on T cells mediates intestinal inflammation and contributes to IgA nephropathy.. J Clin Invest.

[50] Ivanov, II, Frutos Rde L, Manel N, Yoshinaga K, Rifkin DB (2008). Specific microbiota direct the differentiation of IL-17-producing T-helper cells in the mucosa of the small intestine.. Cell Host Microbe.

[51] Ivanov II, Atarashi K, Manel N, Brodie EL, Shima T (2009). Induction of intestinal Th17 cells by segmented filamentous bacteria.. Cell.

[52] Wu HJ, Ivanov II, Darce J, Hattori K, Shima T (2010). Gut-residing segmented filamentous bacteria drive autoimmune arthritis via T helper 17 cells.. Immunity.

